# Role of artificial intelligence in ICU therapeutic decision-making for severe infections

**DOI:** 10.1097/MCC.0000000000001304

**Published:** 2025-07-04

**Authors:** Daniele Roberto Giacobbe, Antonio Vena, Matteo Bassetti

**Affiliations:** a Department of Health Sciences (DISSAL), University of Genoa; b Clinica Malattie Infettive, IRCCS Ospedale Policlinico San Martino, Genoa, Italy

**Keywords:** antimicrobial prescribing, artificial intelligence, intensive care, large language models, machine learning

## Abstract

**Purpose of review:**

To discuss current and future role of artificial intelligence in predicting severe infections and supporting decisions on antibiotic treatment in critically ill patients in intensive care units (ICU), focusing in particular on some relevant conceptual changes compared to classical clinical reasoning.

**Recent findings:**

Several studies have evaluated the ability of machine learning techniques for severe infection prediction, while other studies have explored the potential of large language models (LLM)-based tools to assist clinicians in deciding which antimicrobial agent(s) to prescribe to patients with severe infections.

**Summary:**

The support of artificial intelligence for infection prediction and antimicrobial prescribing has shown the potential to improve the treatment of severe infections in ICU. However, the limited number of studies focused on ICU should be highlighted, along with the need to thoroughly address the issue of patients’ privacy and to improve the ethical and legal frameworks for decision accountability, as well as the transparency and quality of training data. A standardized approach to the accuracy-interpretability trade-off would also be essential to outline a correct and shared approach both for the future conduct of studies and for the interpretation of their evidence for clinical practice.

## INTRODUCTION

In recent years, interest in the potential role of artificial intelligence in improving prediction and antimicrobial treatment of severe infections has rapidly grown [1–3,4^▪^,^5^–^16^].

For example, several studies have evaluated the impact of machine learning techniques for the early diagnosis of sepsis, potentially impacting the timing of antimicrobial treatment through the rapid identification of septic patients, while other studies have explored the potential role of natural language processing (NLP), particularly through the use of large language models (LLM)-based tools, to assist clinicians in deciding which antimicrobial agent(s) to prescribe to patients with severe infections [17,18,19^▪^,^20^,^21^]. This could be of particular interest for the treatment of severe infections in critically ill patients admitted to intensive care units (ICU), a task that typically requires dynamic clinical reasoning involving both rapid decisions on empiric therapy and subsequent modifications or withdrawals of antimicrobial agents based on microbiological test results and disease course [22,23].

In this opinion article, we express our view on the current and future role of artificial intelligence in predicting severe infections and supporting decisions on antibiotic treatment in critically ill patients in ICUs, focusing in particular on some relevant conceptual changes compared to classical clinical reasoning. 

**Box 1:**
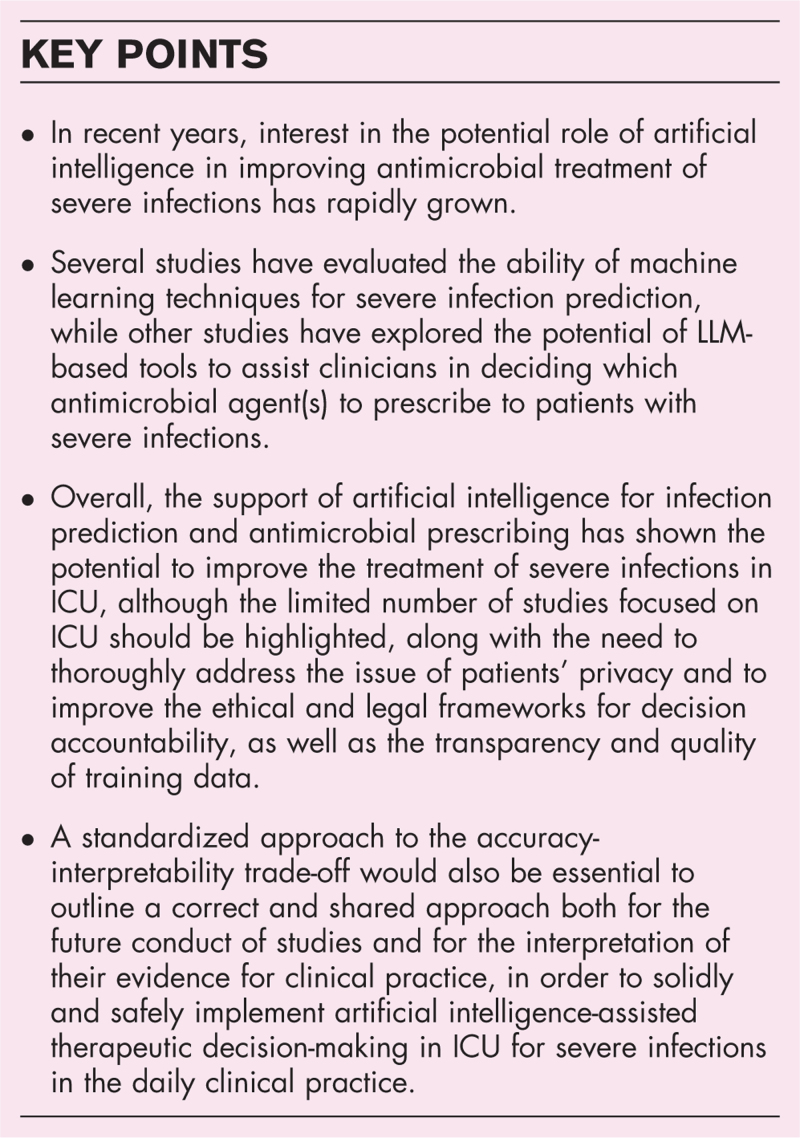
no caption available

## INTELLIGENCE FOR INFECTION PREDICTION AND ANTIMICROBIAL PRESCRIBING; WHEN IS IT ARTIFICIAL?

While there is no universally agreed-upon definition of artificial intelligence yet, one that fits our discussion well is that of machines that can learn from experience, adapt to new inputs, and perform human-like tasks [24–26], with the tasks of interest, for the purposes of this article, being infection prediction and antimicrobial prescribing. In particular, in the context of artificial intelligence, machine learning defines when machines learn tasks from data without being specifically programmed to learn those specific tasks [27]. Thus, can we teach machines to master antibiotic prescribing in critically ill patients simply by feeding them data? Well, the final binary answer is yes, but not without first providing a more complex and comprehensive view, also highlighting the challenges and dangers of indiscriminate use of artificial intelligence without human oversight, at least in the early days of artificial intelligence for clinical decision support.

Starting from the task of infection prediction, the necessary premise is that this task has important implications for the choice of antimicrobial treatment. For example, it is possible to develop models to predict the risk of a specific infection or causative agent (e.g. candidemia, carbapenem-resistant Gram-negative bacteria infection) that, if and when symptoms and signs of severe infection occur, are able to strongly influence physicians’ choices on the type and spectrum of empiric antimicrobial therapy to administer [28–32]. In this context, the first crucial point to discuss is that, in reality, predicting infection by feeding data into models not initially developed for the task of infection prediction is not a novelty for researchers and clinicians. Indeed, many classical models commonly used in the last decades to predict infections in critically ill patients, for example, logistic regression, were commonly used to calculate risks and build scores to predict infection based on collected clinical data [33], and certainly were not developed specifically for infection prediction. Thus, where exactly is the line between classical prediction models and artificial intelligence-based infection prediction (i.e. when is the ‘intelligence’ driving antimicrobial prescribing truly ‘artificial’)? And perhaps more importantly, what conceptual and clinical implications does this difference imply?

## PREDICTION OF INFECTION WITH MACHINE LEARNING TECHNIQUES

Drawing the line between classical and artificial intelligence-based infection prediction studies is far from straightforward. Consider that many introductory lectures in various artificial intelligence and/or machine learning courses for students begin by introducing logistic regression as a machine learning model. Although this may initially confuse clinicians, this is not technically a misclassification, since, as discussed in the previous section, logistic regression ‘learns’ to predict from data. Thus, again, where exactly is the difference between classical and machine learning prediction models? One might be tempted to assume that the latter must be a recent innovation developed to improve the performance of classical prediction models, as the terms artificial intelligence and machine learning have only recently begun to appear prominently in the medical literature. However, this is also not true, as attempts to predict infections with machine learning models (e.g. neural networks, the family of algorithms behind many impressive artificial intelligence technological advances in recent years, including LLM, see next section) date back to more than 20 years ago [34,35]. Two fundamental things have changed dramatically in the last decades, however, the speed of machine computation, which could now make computers able to ‘train’ machine learning models to predict infections using a huge amount of data in a reasonable time; the availability of a large amount of data stored in electronic medical records (EMR), from which to extract clinical variables (or ‘features’ in machine learning terminology) to train machine learning-based infection prediction models, or of unstructured text from the web, including part of the scientific literature, to train LLMs to recognize patterns in text and provide answers to user prompts (for discussion of this latter case, see next section) [36–38].

Regarding infection prediction through machine learning models trained with clinical features, several studies have recently attempted to do so. For example, there are studies aimed at predicting infection by methicillin-resistant *Staphylococcus aureus* (MRSA) or by carbapenem-resistant Gram-negative bacteria [39,40]. An example of a deep learning model (i.e. a neural network with multiple layers) for MRSA infection prediction from EMR data is that of Nigo and colleagues, who extracted EMR data of 8146 MRSA events and 22 393 non-MRSA events obtaining a good predictive accuracy [area under the curve (AUC) 0.911], with subsequent external validation on 5789 MRSA events and 378 924 non-MRSA events (AUC 0.859) [40]. By means of an extreme gradient boosting algorithm, Alparslan and colleagues attempted to predict carbapenem-resistant *Klebsiella pneumoniae* (CRKP) infection among 289 patients with *K. pneumoniae* infection, achieving 83% accuracy, 88% sensitivity, 83% precision, and 85% F1 score [39].

These are just two examples of the many recent efforts in the literature to exploit artificial intelligence and machine learning for infection prediction [26,41–55]. Nevertheless, conceptually they already allow us to highlight some important and peculiar considerations related to the advent of artificial intelligence and machine learning in infection prediction (and in health events prediction in general). The first is that previous prediction studies through classical statistical models focused mainly on explanations rather than accuracy. More in detail, while accuracy/discrimination measures (e.g. standard accuracy and AUC) were also included in previous studies and are essential within a complete evaluation of model performance, the text of the abstracts and the main results of previous studies were dominated by measures explaining which were the characteristics that influenced the prediction [56]. For example, in classic studies evaluating logistic regression-based prediction models, the results were dominated by odds ratios, indicating, for each feature (e.g. a baseline comorbidity, a score of acute phase conditions, the amount of delay in initiating appropriate antibiotic therapy) its impact on the prediction of a given event (e.g. CRKP infection) [28,29]. Although these measures do not indicate causality and only express associations between distributions, they were accessible to clinicians in terms of plausibility within their clinical reasoning (e.g. the association between a delay in appropriate antibiotic therapy and worsening mortality is reasonable from a clinical perspective, considering the worsening of condition and organ function in patients with untreated severe infections, for pathophysiological reasons). In this context, the term ‘explainability’ also does not mean causality. Rather, it expresses the ability to explain which specific characteristics influence the prediction of an event by a given model. However, as discussed above, explanations provide, with all due caution required, a window into plausibility that facilitates clinical understanding and reasoning. Of importance, this could be crucial for rapidly identifying invalid results, aiding in the recognition of biases, and fueling discussions about the reasons behind unexpected associations.

Unike classical prediction studies, although we must admit that there is no published systematic review yet to fully and solidly support this claim, our impression is that recent predictive studies based on machine learning models are more focused, in the main parts of abstracts and results, on accuracy/discrimination rather than explanation [56]. This does not mean that no attempts have been made to provide explanations for machine learning-based prediction models. Indeed, explainable artificial intelligence (XAI) is a growing field of research, aiming to combine the advantages discussed above in identifying which specific factors influence a given prediction [57–60]. The crucial difference, at present, is that, while some classical statistical models such as logistic regression are ‘interpretable’ (i.e. the odds ratios are obtained by exponentiating the regression coefficients of the different features and provide a direct measure of their association with a given outcome measure), some machine learning models (e.g. many neural networks) are not interpretable, as the contribution of a given feature to the prediction cannot be derived from the model's inner calculations [61]. In these cases, XAI is usually implemented through the use of other ‘more interpretable’ machine learning models that attempt to ‘replicate’ the prediction of a more accurate black-box model, thus providing some insights into which features possibly influenced the original prediction by the black-box model [57,62–64]. At least at present, these explanations do not appear to be as reliable as those provided by classical interpretable models, a fact that, in our opinion, may explain the apparent shift from an explanation-based to an accuracy-based approach in reporting the results of infection prediction studies [56,65]. However, the implications are much deeper than simply changing the way study results are presented to readers. In fact, there are two important considerations involved. The first is that whenever an interpretable model performs very similarly to a black-box model, the former should be preferred. This is still the rule in many cases (i.e. having an ‘interpretable’ alternative) for several reasons, one of which may be the current difficulty in automatically extracting granular clinical features rather than administrative codes (not developed for research) from EMR [66]. The role of automation in extraction could indeed become essential, as the very large datasets required for reliable training of many machine learning models inherently imply the inability to manually collect all the necessary data in a reasonable amount of time. However, as NLP techniques improve, it will probably be possible in the future to reliably extract even granular clinical information from EMR automatically and rapidly [67]. If this were to lead to a higher predictive accuracy of black box models without a concomitant sufficient improvement of XAI techniques, this would also mean a major shift in the approach, by clinicians, to the interpretation and use of the results of predictive studies, which should take into account the possibility of a partial or total lack of clarity on how a model reached a given prediction [68]. Although this certainly represents a disadvantage, the ability to identify complex predictive patterns of features impossible for humans to recognize (moving, conceptually, the black box from *a posteriori* to *a priori*, in the latter case as an intended advantage and not as a limitation) could contrarily be useful from the perspective to improve our ability to predict infection [69,70]. Whether there will be an alternative to this in the future will depend on the pace of progress in rapid diagnostic techniques for severe infections and their cause, as, notably, the availability of etiological diagnosis from or very close to the onset of infection would eliminate the need to use predictions as early diagnostic tools while waiting for diagnostic results. At the same time, the issue of the accuracy-interpretability trade-off would be shifted from diagnosis to prevention, that is, when predictive models are used to identify a high risk of developing an infection before the development (and diagnosis) itself, a scenario in which reliable explainability could also be crucial to identify modifiable risk factors that can be addressed to reduce the risk of infection.

## THE ADVENT OF LARGE LANGUAGE MODELS FOR SUPPORTING ANTIMICROBIAL PRESCRIBING

The availability of large corpora of unstructured data (e.g. text from the web, including some of the scientific literature) and advances in NLP models, especially LLM, have recently led to a rapid increase in the global use of LLM-based chatbots that allow users to ask questions (including summarizing large amounts of information) and receive, within seconds, fluent and articulated answers in natural human language [19^▪^,^71^]. In this regard, a crucial breakthrough occurred in 2017 with the introduction of transformers, neural network models that rely on the self-attention mechanism and can handle long-range text dependencies [72]. From a technical perspective, LLMs predict subsequent tokens (i.e. words or parts of words) in a sentence [19^▪^].

Overall, the potential of this technology is certainly huge, as it could help accelerate and improve a broad spectrum of tasks related to the treatment of severe infections in the ICU, from ‘low-risk’ tasks (e.g. quickly summarizing for clinicians the guidelines and relevant literature on the treatment of a given infection) to ‘high-risk’ tasks (e.g. suggesting a given antibiotic or antifungal for a given patient) [73,74^▪▪^]. However, there are still many limitations to the adoption of LLM-based tools, both generic and domain-specific, for antimicrobial prescribing and for healthcare tasks in general. Indeed, current research efforts have demonstrated a still suboptimal accuracy, the presence of large heterogeneity among responses, and the still unacceptable rate of hallucinations (i.e. when chatbots generate seemingly coherent and authoritative text that is, in reality, invented or factually incorrect) in some scenarios [18,21,73,74^▪▪^,^75^–^79^]. Of course, all these issues are likely to be alleviated with future technological advances (e.g. but not limited to, retrieval augmented generation techniques), although probably in a nonlinear way: for example, the rate of hallucinations in specific domains may sometimes increase during model improvements [74^▪▪^,^80^–^84^]. However, the above only represents a part of the challenges related to the implementation of LLM support in healthcare. For example, the black box nature of LLM, as for artificial intelligence-based infection prediction (see previous section), introduces other peculiar aspects that need to be carefully addressed before LLM-based tools are widely adopted in healthcare (to the best of our knowledge, no LLM-based tool has been licensed for use in daily clinical practice so far). On the other hand, some healthcare systems have started to implement LLM-based tools for use by clinicians, possibly integrated into EMR [74^▪▪^,^85^]. While this is in line with the enormous potentials of LLMs in terms of future support to physicians for antimicrobial prescribing, it also further highlights the absolute need for concomitant progress in addressing all relevant issues related to the future routine use of this technology, including not only privacy and liability issues, which require the development of dedicated ethical and legal frameworks, but also the need for educational efforts to train clinicians and future clinicians to recognize the functioning of LLM and the related implications in terms of biases and omissions, which would need to be carefully taken into account during any future clinical reasoning supported by LLM-based tools.

**FIGURE 1:**
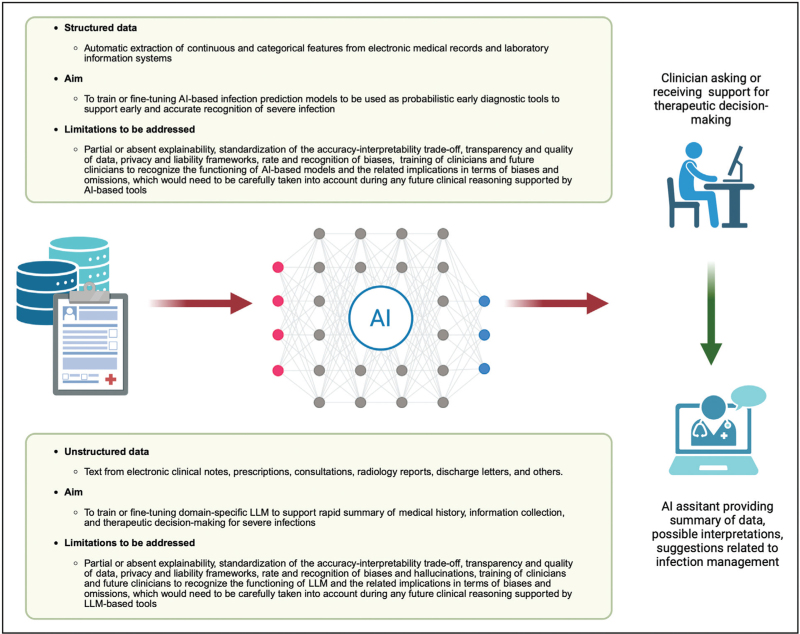
The possible future of infection prediction and artificial intelligence-based support in ICU therapeutic decision-making for severe infections. AI, artificial intelligence; LLM, large language models. Created in BioRender. Giacobbe DR (2025). https://BioRender.com/qqrifu7.

A graphical summary of the topics covered in the article relating to the role of artificial intelligence in ICU therapeutic decision-making for severe infections is available in Fig. 1.

## CONCLUSION

The support of artificial intelligence for infection prediction and antimicrobial prescribing has shown the potential to improve the treatment of severe infections in ICU. However, the limited number of studies focused on ICU should be highlighted, along with the need to thoroughly address the issue of patients’ privacy and to improve the ethical and legal frameworks for decision accountability, as well as the transparency and quality of training data. Finally, a standardized approach to the accuracy-interpretability trade-off, when needed (this trade-off is indeed not an absolute rule, it depends on the scenario and tasks), would also be essential to outline a correct and shared approach both for the future conduct of studies and for the interpretation of their evidence for clinical practice, in order to solidly and safely implement artificial intelligence-assisted therapeutic decision-making in ICU for severe infections in the daily clinical practice.

## Acknowledgements

*None.*

### Financial support and sponsorship

*None.*

### Conflicts of interest

*Outside the submitted work, M.B. has received funding for scientific advisory boards, travel, and speaker honoraria from Cidara, Gilead, Menarini, MSD, Mundipharma, Pfizer, and Shionogi. Outside the submitted work, D.R.G. reports investigator-initiated grants from Pfizer, Shionogi, bioMérieux, Menarini, Tillotts Pharma, and Gilead Italia, travel support from Pfizer, and speaker/advisor fees from Pfizer, bioMérieux, Advanz Pharma, Menarini, and Tillotts Pharma. A.V. has no conflicts of interests to disclose.*

## REFERENCES AND RECOMMENDED READING

Papers of particular interest, published within the annual period of review, have been highlighted as:

▪ of special interest
▪▪ of outstanding interest
